# Intracoronary Optical Coherence Tomography: Technological Innovations and Clinical Implications in Cardiology

**DOI:** 10.1007/s11936-025-01103-4

**Published:** 2025-07-24

**Authors:** Yuchen Jiang, Jingyi Wang, Raksha Sreeramachandra Murthy, Pranav Patel, Zhongping Chen

**Affiliations:** 1 Beckman Laser Institute, University of California Irvine, Irvine, CA 92612 USA; 2 Department of Biomedical Engineering, University of California Irvine, Irvine, CA 92612 USA; 3 Department of Electrical Engineering and Computer Science, University of California Irvine, Irvine, CA 92612 USA; 4 Edwards Lifesciences Foundation Cardiovascular Innovation and Research Center, University of California, Irvine, CA 92697 USA; 5 Division of Cardiology, Irvine Medical Center, University of California, Orange, CA 92868 USA

**Keywords:** OCT, Multimodality Imaging, Cardiology, Complex Lesions, Optical Coherence Elastography

## Abstract

**Purpose of Review:**

To provide the most up-to-date clinical evidence of intracoronary optical coherence tomography (OCT), and clinical implications to guide future imaging research in cardiology.

**Recent Findings:**

Intracoronary OCT has demonstrated advanced system performance and high reproducibility in analyzing atherosclerotic lesions. It is an attractive tool due to its capability for functional classification and superior imaging resolution, enabling precise and reliable tissue assessments. Compared to traditional angiography, OCT has been associated with improved long-term clinical outcomes and serves as an effective tool for optimizing stent selection and post-intervention evaluation. The development of OCT variations and the combination of various intravascular imaging modalities further enhance its diagnostic capabilities, allowing a comprehensive assessment of complex vulnerable lesions and improving risk stratification for patients.

**Summary:**

Current and evolving system development presents a hopeful path for treating coronary artery disease by addressing the challenges of the intracoronary OCT technique. Future studies focusing on utilizing OCT system extensions, integrated multimodality imaging systems, and Artificial Intelligence (AI) derived image analysis will improve clinical endpoints and streamline the process.

## Opinion Statement

Intracoronary cardiac imaging plays an important role in optimizing interventional success, particularly for patients undergoing balloon angioplasty and stent implantation. Among intracoronary imaging modalities, intravascular optical coherence tomography (IVOCT) stands out as a powerful tool, providing unparalleled high-resolution visualization of coronary artery pathology. To bridge the gap in clinical translation, researchers are exploring various technological directions. Some focus on functional OCT techniques, such as polarization-sensitive OCT (PS-OCT) and optical coherence elastography (OCE), which provide additional tissue birefringence and strain information. Others have been working on miniaturizing imaging catheters that integrate multiple intravascular imaging modalities. Each imaging modality offers distinct advantages: intravascular ultrasound (IVUS) provides deep penetration, enabling full visualization of the artery wall; intravascular photoacoustic imaging offers volumetric data and chemical composition information in deep tissues; intravascular elastography maps the artery by measuring the tissue strain and mechanical properties in response to physiological forces such as blood pressure; intravascular PS-OCT enhances tissue contrast between fibrous, lipid-rich and calcified plaques by analyzing birefringence. Researchers propose that integrating these different imaging techniques enables a more comprehensive characterization of plaques. Recently, many multimodality imaging systems patents have been granted, alongside FDA approvals for multimodal imaging devices such as IVUS-OCT catheter. These developments mark a new revolutionary stage of cardiac imaging. Additionally, educating healthcare professionals on the use of intracoronary OCT is essential for reducing cardiac events rates. The accelerated development of AI-driven platforms is transforming plaque complexity analysis by enhancing image interpretation, improving workflow efficiency, and expanding accessibility. These advancements will significantly expand the reach of intravascular imaging systems in the future.

## Introduction

Coronary artery disease (CAD) is one of the leading causes of death in the United States [1]. Angiography has been the primary imaging modality in percutaneous coronary intervention (PCI), serving as the gold standard for visualizing coronary anatomy and guiding revascularization procedures. This technique relies on the administration of a radiopaque contrast agent, followed by X-ray fluoroscopy. However, angiography is limited to intraluminal and arterial wall structure information [2–4]. To address this problem, IVUS was introduced for plaque morphology assessment. However, it is constrained by a relatively low spatial resolution of around 100–300 μm, limiting the detailed visualization of microstructural features [5–8].

To overcome these limitations, intracoronary OCT was developed as a high-resolution, catheter-based imaging modality. OCT is a non-invasive imaging technology which utilizes back-scattered infrared light to produce a two-dimensional image of tissue internal microstructures. It enables high-resolution, real-time, cross-sectional visualization of biological tissues, making it a valuable tool in diagnostic and therapeutic applications. Initially developed for ophthalmic imaging, OCT has played a crucial role in the early detection of retinal diseases such as glaucoma and macular degeneration. Over the past decades, its applications have expanded beyond ophthalmology to diverse medical fields, including neurology, dermatology, gynecology, and cardiology [9–12]. In recent years, it has shown a high impact on coronary artery disease, in both disease diagnosis and treatment planning [13].

It provides detailed visualization of arterial wall morphology and offers complementary diagnostic insights beyond conventional angiography. Clinical studies have demonstrated the efficacy of OCT-guided interventions, supporting the systems’ reliability in interventional cardiology [14–16]. However, the adoption of OCT guidance in routine clinical practice remains limited, primarily due to challenges in image interpretation and the need for further large-scale studies to validate its impact on clinical outcomes [17].

To fill the gap between benchtop and bedside for OCT, researchers have been investigating multiple strategies to enhance its role in diagnosis, risk assessment, and prevention of cardiovascular events. In this review, we present a detailed overview of intracoronary OCT, highlighting recent advancements. Additionally, we discuss novel integrations of OCT with other functional intravascular imaging modalities and introduce emerging AI-based quantitative analysis techniques in intracoronary imaging.

## Development of Intracoronary OCT

### OCT: Advanced Pathway, Acquisition Steps, Limitations

#### Advanced Pathway

Intracoronary OCT has gained wide attention in coronary artery diseases, particularly atherosclerosis [18]. Multiple intracoronary OCT imaging systems have been launched on the market over the past few years [19–21]. The implementation of OCT as a guidance technique during PCI treatment gained significant attention in clinical studies [22]. The CALIPSO trial was a pioneering randomized controlled study that compared OCT-guided PCI to angiography-guided PCI in patients with moderate to severe calcified lesions [23]. Findings indicated that OCT guidance led to greater post-PCI outcomes, underscoring its potential advantages in complex lesion management. In addition, studies have demonstrated that OCT-guided PCI achieves clinical outcomes comparable to IVUS, with both modalities offering excellent reproducibility [24–26].

As an imaging guidance tool, OCT facilitates precise pre-procedural assessment of complex lesions and enhances stent deployment accuracy, thereby reducing post-PCI complications [27]. With rapid image acquisition rates, intracoronary OCT systems can perform a single pullback scan over a 72 mm vessel segment in approximately 2.7 s, significantly minimizing motion artifacts [28].

#### Imaging Protocol

An intravascular OCT imaging system comprises an imaging catheter, a motorized control unit, and acquisition software for image capture and reconstruction. The integrated system enables high-speed imaging over 100 frames per second (fps), with a pullback speed of around 20–40 mm/s. A standard system typically features a rigid distal tip of approximately 4 mm, an axial resolution of ~10–15 μm, a lateral resolution of ~25 μm, and a~10 mm diameter field of view [29].

During a standard coronary intervention treatment, OCT imaging is typically performed twice: once before stent deployment to aid in lesion assessment and stent sizing, and again post-intervention to optimize stent placement and evaluate procedural outcomes. A minimum 6-French guiding catheter is required to advance the OCT catheter into the coronary arteries. Access is typically obtained via the femoral or brachial artery. A standard 0.014-inch guidewire is then advanced across the lesion under angiographic guidance [30]. The guiding catheter must be properly positioned at the coronary ostium to provide adequate support for the imaging catheter. Standard OCT imaging catheters have an outer diameter of approximately 3-French, a working length of around 135 cm, and an imaging penetration depth of 1–3 mm. The catheter is advanced over the guidewire into the coronary artery, with the imaging segment positioned distal to the target plaque. Once correctly positioned, the pullback scan is initiated, and the data collection and reconstruction begin. Each OCT frame can be acquired with 1,000 A-lines, and with a 100 kHz laser, the imaging speed can reach 100 fps. For a 50 mm artery, at a pullback speed of 20 mm/s, the entire artery can be scanned in just 2.5 s. The collected images allow physicians to evaluate vessel morphology, plaque characteristics, and stent apposition in real-time.

Specialized catheters designed for small vessels feature a reduced outer diameter of less than 3-French, allowing imaging in narrowed lumens. In clinical practice, each OCT scan typically requires 10–15 mL of a non-ionic contrast agent to clear blood from the imaging field [31].

#### Limitations

Recent comparison studies have shown the capability of intracoronary OCT to acquire precise cross-sectional images of arterial walls. However, OCT faces several challenges, particularly for patients with diabetes, who frequently present with complex vulnerable lesions. While OCT can achieve high resolution in superior lesion layer assessment, its depth limitation challenges the visualization of underlying structures. In cases where the top calcified layer is thick, OCT may not be able to detect lipid or calcium deposits in deep tissue, restricting its diagnostic utility in such scenarios. Many recent studies have focused on advancing the OCT technique by extending the concept of OCT such as building a polarization-sensitive OCT system to solve this problem [32]. Additionally, research has shown that the integration of multimodal imaging combining intracoronary OCT with IVUS, or angiography could provide more comprehensive vessel characterization, enhancing model robustness and clinical relevance [33–35].

Moreover, AI and software modeling have expanded rapidly in the field of plaque analysis for intracoronary OCT [36–38]. Before AI was introduced, plaque analysis relied on cardiologists manually interpreting imaging data, requiring a subjective and time-consuming assessment of plaque buildup in coronary arteries. With the emergence of AI-powered algorithms, particularly deep learning techniques, plaque detection, characterization, and risk assessment can now be fully automated, significantly improving efficiency and reducing interobserver variability [39].

### Intracoronary polarization-sensitive OCT (PS-OCT)

PS-OCT is an advanced OCT modality that utilizes the polarization properties of light to extract birefringence information from biological tissues. Unlike conventional intracoronary OCT, which primarily detects light intensity variations, PS-OCT captures changes in the polarization state of light, providing additional contrast based on tissue microstructure [40]. Intracoronary PS-OCT has demonstrated its capability to identify birefringence signals corresponding to collagen-rich regions within the plaques. This enables a more precise evaluation of fibrous cap thickness, lipid core composition, and inflammatory markers, which are critical for identifying high-risk plaques and optimizing cardiovascular interventions [41].

**Fig. 1:**
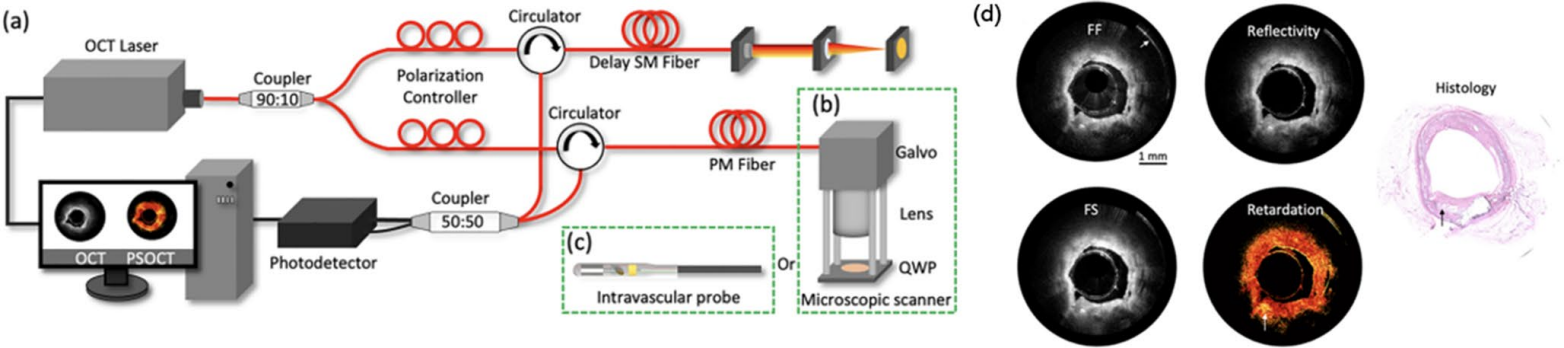
(**a**) the schematic of the IV-PSOCT system, (**b**) the microscopic scanner, (**c**) the intracoronary imaging probe, and (**d**) the retardation and histology images of cadaver coronary arteries. (Adapted from Scientific Reports. 2022;12(1):6831; https://www.nature.com/articles/s41598-022-10709-8; Creative Commons user license https://creativecommons.org/licenses/by/4.0/) [41]

Intracoronary PS-OCT shows great potential as an imaging modality, particularly in assessing the biochemical properties of arterial tissues. Retardation images, generated by PS-OCT systems, visualize the cumulative phase shift (retardation) between orthogonal polarization states of light as it propagates through birefringent tissues. These images provide functional contrast based on the differences in collagen content [42]. In Fig. 1d, the birefringence effect indicated by the black arrow can be found, which corresponds to cholesterol crystals. The results clearly demonstrate different levels of birefringence inside the tissue, highlighting the system’s ability to distinguish vulnerable plaques. Further advancements can quantitatively analyze techniques, such as birefringence decomposition and collagen density estimation. Besides this, optimizing PS-OCT imaging catheters remains essential for conducting in vivo human studies, which are prerequisite for clinical translation. The curvature of the catheter frequently induces changes in the polarization state within the optical fiber during rotation, potentially leading to inaccurate polarization measurements and erroneous outcomes. Several studies, including those by Villiger et al. (2024), have focused on overcoming those challenges. Their proposed new reconstruction method with catheter-based PS-OCT system successfully mitigates the limitations posed by dynamic catheter rotation, providing a more reliable and robust measurement of artery tissue [43]. In addition, Villiger et al. (2025) also presented a new postprocessing pipeline utilizing intravascular PS-OCT images to efficiently measure plaque properties including fibrous cap thickness [44]. These advancements represent a critical step toward the clinical translation of catheter-based PS-OCT imaging. Recent studies have also explored combining PS-OCT with other imaging modalities. Julien Bec et al. recently integrated PS-OCT with fluorescence lifetime imaging (FLIm) and developed a dual modality intravascular catheter. This novel system acquired biochemical composition by FLIm, and tissue birefringence mapping from the PS-OCT system, making it a suitable device for plaque detection [45].

#### Multimodality Systems

Multimodal imaging systems have emerged as a significant area of research in intracoronary imaging. OCT has been integrated with various imaging modalities to enhance the quantitative assessment of atherosclerotic plaques, including IVUS and intravascular photoacoustic imaging (IVPA) [46–50]. The multimodality approaches improve plaque characterization by providing detailed structural, compositional, and biomechanical insights beyond what individual modalities can achieve alone.

*IVUS-OCT*.

IVUS was developed as a minimally invasive imaging technique and is commonly employed in clinical practice. It offers deep tissue penetration but with moderate spatial resolution. The integration of IVUS and OCT has garnered significant public interest, as it is expected to achieve both high surface resolution and deep structure anatomy of plaque lesions [51].

**Fig. 2:**
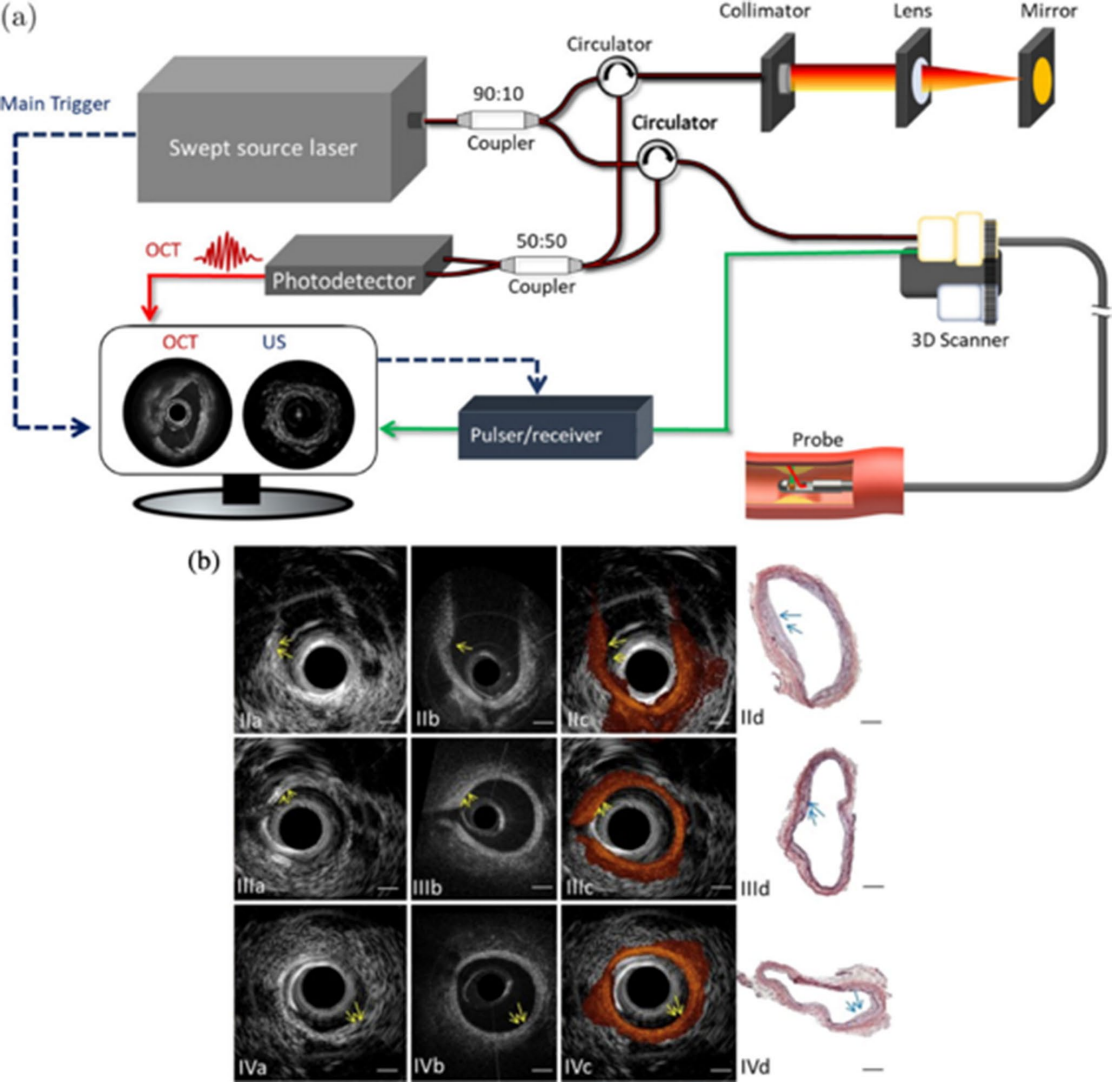
Integrated IVUS-OCT. (**a**) A schematic of dual-modality IVUS and OCT system. (**b**) OCT, IVUS, and histology of calcified, lipid-rich, and fibrous plaques. (Adapted from Scientific Reports. 2015;5(1):18406; https://www.nature.com/articles/srep18406; Creative Commons user license https://creativecommons.org/licenses/by/4.0/) [52]

Figure 2 shows an imaging system integrating OCT and IVUS. The co-registered image helps further characterize different plaque pathologies. IVUS provides structural visualization of the arterial wall, where the intimal thickening and low-density acoustic regions indicate plaque presence. However, IVUS cannot differentiate between plaque types. In corresponding OCT images, a homogeneous boundary with a weak signal beneath a high-intensity region suggests a lipid-rich plaque (Fig. 2 IIIb). Histological analysis (Fig. 2 IIId) confirms these findings.

IVUS-OCT imaging technology has undergone significant development in clinical research. Following recent FDA regulatory clearance, the integration of IVUS and OCT modalities has facilitated comprehensive imaging of human coronary arteries [53–56]. The PANOVISION trial rigorously evaluated the clinical performance of a hybrid intravascular ultrasound-optical coherence tomography imaging system in patients undergoing coronary interventions [57]. The findings demonstrated that the dual modality system provided high-quality imaging without procedure-related adverse cardiovascular events, underscoring its safety and potential clinical utility in future cardiovascular research.

*IVOCT-US-PA system*.

Photoacoustic signals can detect the intra-lesion lipid density and the cholesterol content, which correlate with the plaque complexity and rupture risk. Unlike OCT or IVUS, IVPA images provide volumetric data, enabling the ability to image the whole arterial wall. Moreover, IVUS primarily provides mostly structural information, whereas IVPA offers molecular contrast in deep tissue.

**Fig. 3:**
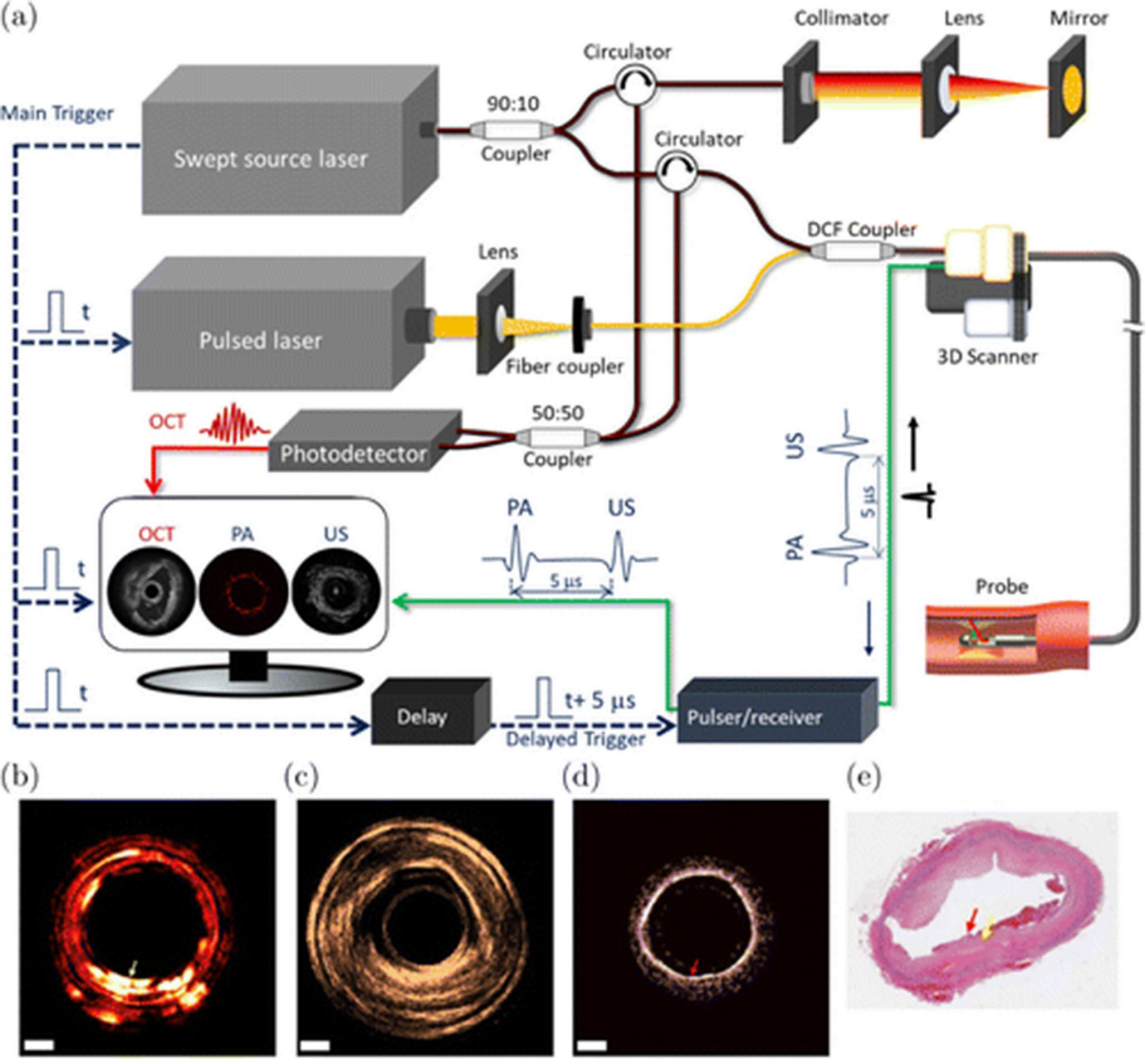
Integrated IVOCT-US-PA. (**a**) Tri-modality system design. (**b**) PA (c) IVUS (**d**) OCT and (**e**) Histology of ex-vivo rabbit aorta. (Adapted with permission from Dai X, et al. Acs Photonics. 2017;4(1):174−80. Copyright © 2017) American Chemical Society) [58]

Figure 3(a) shows the system schematic. Ex-vivo experiment results obtained from a tri-modality IVOCT-US-PA system were presented in Fig. 3 (b) to (e) [58]. The feasibility of the photoacoustic imaging in depth-resolved tissue composition was demonstrated. In addition to that, Wang et al. (2024) recently developed a dual-mode intravascular imaging system combining photoacoustic and OCT to detect spontaneous coronary artery dissection [59]. The integrated system successfully identifies the key features, including intimal tears and intramural hematomas, demonstrating its potential for enhanced diagnosis and management of this condition.

Optical coherence elastography, another functional extension of OCT, has been widely used as a powerful tool for tissue imaging and therapeutic applications [60–62]. Several approaches have been explored for endoscopic OCE [63]. By utilizing the acoustic radiation force, an ARF-OCE imaging probe was developed. Its capability was validated in quantifying tissue mechanical properties and differentiating diseased tissue from healthy tissue [64]. The circumferential intravascular OCE images were successfully obtained by Wang et al. (2022) at a imaging speed of 3019 frames per second for the first time, enabling a simultaneously mapping both the artery tissue structure and stiffness [65]. This novel catheter provides enhanced detection of calcifications in atherosclerotic plaques. Additionally, Xu et al. (2024) demonstrate a miniaturized 0.9 mm outer diameter OCE probe capable of performing the first elasticity imaging in vivo rat vagina, providing strong evidence for clinical translation in the future [66].

Multimodality systems overcome the limitation of intracoronary OCT as a single modality imaging technology by providing more information on plaque characteristics. An ideal intracoronary imaging catheter should provide determining factors including morphology, composition, and mechanical properties of lesions. Therefore, a high-speed intracoronary PS-OCT-US-PA imaging system holds great potential to provide a more quantitative comprehensive evaluation of complex vulnerable plaques.

### Plaque Assessment with AI

Intracoronary OCT plays a key role in cardiovascular interventions. However, it generates large volumes of data that require manual interpretation, making the process labor-intensive and prone to variability and subjectivity. AI has emerged as a powerful tool to automate and enhance image analysis, improving accuracy, efficiency, and clinical decision-making. This section reviews existing technologies and explores future directions for AI-assisted plaque assessment, highlighting its potential to improve clinical interventions. The development of AI in intracoronary OCT images has progressed through several key stages, each addressing specific challenges in vascular imaging and analysis.

#### Segmentation of Vascular Structures

The initial focus was on segmenting vascular structures, such as the lumen and vessel walls, to facilitate quantitative analysis. Early AI models, particularly convolutional neural networks (CNNs) like U-Net, were employed to delineate these structures accurately [67]. To enhance segmentation performance, various hyperparameters were optimized, including the selection of appropriate loss functions and the implementation of data augmentation techniques [68–70]. These strategies have been instrumental in improving the accuracy and robustness of vascular structure segmentation in OCT imaging.

#### Plaque Characterization and Classification

Following segmentation, AI models were developed to classify different types of plaques and tissue components. Advanced architectures, such as ResNet and Vision Transformer (ViT)-based models, were utilized to improve classification accuracy [71–74]. These models enabled the identification of vulnerable plaques, which are critical for assessing the risk of adverse cardiovascular events. For example, AI-driven analysis has been used to detect plaque erosion in coronary arteries, a condition associated with increased risk of thrombosis. Researchers also developed models capable of distinguishing various plaque components, such as fibrous, lipid-rich, and calcified tissues, thereby enhancing diagnostic accuracy and reducing the subjectivity associated with manual interpretation [75, 76]. Specifically for fibrous lesions, the thickness of the fibrous cap overlying atherosclerotic plaques is a critical determinant of plaque stability. AI-driven approaches have been utilized to automate the segmentation and measurement of fibrous cap thickness in IVOCT images, providing valuable insights into plaque stability and aiding risk stratification [77–80]. These advancements have been instrumental in improving the objectivity and reproducibility of IVOCT.

#### Quantitative Analysis and Risk Assessment

AI advancements have enabled quantitative analysis of vascular features, such as plaque burden, vessel diameter, stent analysis, and wall thickness [81]. This quantitative approach aids in assessing the severity of coronary artery disease and evaluating the risk of future cardiovascular events. Studies have shown that AI-enhanced OCT can assist cardiologists in diagnosing coronary plaque vulnerability [82–84].

Despite significant advancements in deep learning for IVOCT, several challenges remain that hinder its full clinical integration. One major limitation is the dependency on large, well-annotated datasets for training. Given the time-consuming and expertise-dependent nature of manual annotation, publicly available datasets remain limited. Another challenge is the lack of interpretability and clinical trust in deep learning models. While deep learning achieves high accuracy in vessel wall segmentation, its decision-making process remains largely a “black box” for clinicians. Furthermore, existing models often struggle with high-attenuating plaques, such as lipid-rich or heavily calcified regions, leading to inconsistencies in interpretation. Real-time processing constraints, particularly in resource-limited settings, further hinder the deployment of AI models in clinical workflows.

#### Future Directions of AI

Future research should focus on developing more data-efficient deep learning methods, such as self-supervised learning, semi-supervised learning, and meta-learning, to reduce dependency on large, annotated datasets. Transfer learning from related imaging modalities could also help improve generalizability. Improving model interpretability remains a key priority, with explainable AI (XAI) techniques offering potential solutions for increasing clinician trust. Methods such as attention maps, feature visualization, and uncertainty quantification could provide insights into model predictions, facilitating better clinical decision-making. Another promising direction is the development of real-time AI models optimized for deployment in catheter-based imaging systems, enabling on-the-fly analysis during procedures. Finally, larger-scale clinical validation studies and regulatory approvals are crucial to transitioning deep learning-based IVOCT analysis from research settings to routine clinical practice. Establishing standardized evaluation metrics and benchmarking datasets will be essential for ensuring reproducibility and reliability across different healthcare institutions. By addressing these challenges, deep learning has the potential to revolutionize IVOCT analysis, improving diagnostic precision, procedural efficiency, and patient outcomes.

## Conclusions

Intracoronary OCT is a promising clinical tool for understanding vessel wall composition, disease stage assessments, and PCI guidance. Intracoronary PS-OCT and intravascular multimodality systems have demonstrated great potential in characterizing atherosclerotic plaques. As multimodality imaging emerges as a promising direction for clinical studies, the recent FDA clearance of IVUS/OCT imaging catheters is expected to accelerate their adoption, expanding clinical applications and transforming cardiovascular research. In addition, intracoronary OCE offers detailed information about arterial wall strain, making it a powerful tool for studying coronary diseases in the future. Finally, AI-integrated image analysis provides a quantitative approach for evaluating vascular features. AI technology has advanced significantly, providing robust evidence for plaque risk assessment and enhancing cardiologists’ clinical decision-making.

## Key References

- Jin Q, Fu Z, Wang Y, Zeng Y, Zhang X, Ye Y, et al. A Multicenter Feasibility and Safety Study of a Novel Hybrid IVUS-OCT Imaging System. JACC: Asia. 2025;5(3_Part_1):396-400.
  - This study demonstrates the ability of the multimodal imaging catheter in clinical applications.
- Bec J, Zhou X, Villiger M, Southard JA, Bouma B, Marcu L. Dual modality intravascular catheter system combining pulse-sampling fluorescence lifetime imaging and polarization-sensitive optical coherence tomography. Biomed Opt Express. 2024;15(4):2114-32. doi: 10.1364/BOE.516515.
  - This study demonstrates the ability of the multimodal imaging catheter in clinical applications.
- Wang T, Pfeiffer T, Akyildiz A, van Beusekom HMM, Huber R, van der Steen AFW, et al. Intravascular optical coherence elastography. Biomed Opt Express. 2022;13(10):5418-33. doi: 10.1364/BOE.470039.
  - This study successfully obtained the first circumferential intravascular OCE images of artery tissues, providing both structural and stain information.

## Data Availability

No datasets were generated or analysed during the current study.
